# Decreased annual risk of tuberculosis infection in South Korean healthcare workers using interferon-gamma release assay between 1986 and 2005

**DOI:** 10.1186/s12879-021-06855-5

**Published:** 2021-11-16

**Authors:** Eun Hye Lee, Nak-Hoon Son, Se Hyun Kwak, Ji Soo Choi, Min Chul Kim, Chang Hwan Seol, Sung-Ryeol Kim, Byung Hoon Park, Young Ae Kang

**Affiliations:** 1 Division of Pulmonology, Allergy and Critical Care Medicine, Department of Internal Medicine, Yongin Severance Hospital, Yonsei University College of Medicine, Yongin, Gyeonggi-do Republic of Korea; 2 Center for Digital Health, Yongin Severance Hospital, Yonsei University College of Medicine, Yongin, Gyeonggi-do Republic of Korea; 3 Data Science Team (Biostatistician), Center for Digital Health, Yongin Severance Hospital, Yonsei University College of Medicine, Yongin, Gyeonggi-do Republic of Korea; 4 Division of Pulmonary and Critical Care Medicine, Department of Internal Medicine, Severance Hospital, Yonsei University College of Medicine, 50-1 Yonsei-ro, Seodaemun-gu, Seoul, 03722 Republic of Korea; 5 Institute of Immunology and Immunological Diseases, Yonsei University College of Medicine, 50-1 Yonsei-ro, Seodaemun-gu, Seoul, 03722 Republic of Korea

**Keywords:** Latent tuberculosis infection, Prevalence, Annual risk of infection, Interferon-gamma release assays, Health care workers

## Abstract

**Background:**

Tuberculosis (TB) has been a major public health problem in South Korea. Although TB notification rate in Korea is gradually decreasing, still highest among the member countries of the Organization for Economic Cooperation and Development. To effectively control TB, understanding the TB epidemiology such as prevalence of latent tuberculosis infection (LTBI) and annual risk of TB infection (ARI) are important. This study aimed to identify the prevalence of LTBI and ARI among South Korean health care workers (HCWs) based on their interferon-gamma release assays (IGRA).

**Methods:**

This was single center, cross-sectional retrospective study in a tertiary hospital in South Korea. We performed IGRA in HCWs between May 2017 and March 2018. We estimated ARI based on IGRA results. Logistic regression model was used to identify factors affecting IGRA positivity.

**Results:**

A total of 3233 HCWs were analyzed. Median age of participants was 38.0 and female was predominant (72.6%). Overall positive rate of IGRA was 24.1% and IGRA positive rates age-group wise were 6.6%, 14.4%, 34.3%, and around 50% in the age groups 20s, 30s, 40s, and 50s and 60s, respectively. The ARIs was 0.26–1.35% between 1986 and 2005; rate of TB infection has gradually decreased in the last two decades. Multivariable analysis indicated that older age, healed TB lesion in x-ray, and male gender were risk factors for IGRA positivity, whereas working in high-risk TB departments was not.

**Conclusions:**

Results showed that ARI in South Korean HCWs gradually decreased over two decades, although LTBI remained prevalent. Our results suggest that the LTBI test result of HCWs might be greatly affected by age, rather than occupational exposure, in intermediate TB burden countries. Thus, careful interpretation considering the age structure is required.

**Supplementary Information:**

The online version contains supplementary material available at 10.1186/s12879-021-06855-5.

## Background

South Korea is an intermediate tuberculosis (TB) burden country; in 2019, the annual incidence of TB was reported to be 59 per 100,000 [1]. TB is still an important public health concern in South Korea; for effective TB control, understanding its epidemiology, such as the prevalence of latent tuberculosis infection (LTBI) and annual risk of TB infection (ARI) is important. ARI represents the proportion of newly infected or re-infected people over a 1-year period calculated based on the prevalence of LTBI. LTBI prevalence has steadily decreased in South Korea [2, 3]; however, about one third of the general population in South Korea is still thought to have LTBI [4, 5].

To date, LTBI prevalence studies and analysis of ARI have been conducted mainly using the tuberculin skin test (TST) in South Korea [2, 6, 7]. In previous studies, the annual risk of TB infection in South Korea was estimated using TST results in ages 5–9 years, and the ARI was 0.5–5.3% based on a 10-mm induration between 1958 and 1988 [6]. However, studies on ARI using interferon-gamma release assay (IGRA) results in South Korea are lacking.

In this study, we aimed to identify the prevalence of LTBI and estimate ARI in health care settings based on IGRA results of HCWs. We also aimed at identifying the risk factors of LTBI for HCWs with a particular focus on their age in an intermediate TB burden country.

## Methods

### Study population

This study was conducted at Severance Hospital, a 2500-bed tertiary referral hospital in South Korea. A total of 3283 HCWs underwent the LTBI test between May 2017 and March 2018 as part of a national TB elimination program. The LTBI test used was QuantiFERON® TB-Gold In Tube test (QFT-GIT). High-risk departments such as respiratory department and outpatient clinic, medical intensive care unit, emergency department, microbiology laboratory, and radiology department were defined as TB-related departments. We collected data related to participants’ age, sex, occupation, hospital department, and years of employment; other electronic medical records were retrospectively reviewed. Previously healed TB lesions on chest radiographs were defined as calcified nodules or lymph nodes, discrete fibrotic scars with volume loss, or pleural thickening or calcification [8, 9]

### IGRA

The IGRA was performed using a QuantiFERON® TB-Gold In Tube test (QIAGEN, Hilden, Germany) following the manufacturer’s instructions. A positive IGRA result was defined as an interferon-gamma response to the TB antigen minus that of the nil tube of ≥ 0.35 IU/mL. Chest radiographs were evaluated to exclude active TB at the same period. The HCWs were diagnosed with LTBI if the IGRA results were positive.

### Annual risk of tuberculosis infection (ARI)

The study subjects were divided into nine age groups (20–24, 25–29, 30–34, 35–39, 40–44, 45–49, 50–54, 55–60, and 60–69 years). ARIs were calculated based on the results of IGRAs using the following math formula [10]

- ARI (%) = (1 − [1 − IGRA positive rate of each age group/100]^[1/mean age of each age group]^) × 100
- Midyear of TB infection (y) = 2017-mean age of each age group/2

### Statistical analysis

Continuous variables were presented as means ± standard deviation (SD), or median and interquartile range (IQR), and were analyzed using the Student *t*-test or the Mann–Whitney test. Categorical variables were reported as number and percentage, and were analyzed using the chi-square test or Fisher’s exact test. To determine if IGRA positivity differed according to age in this study, we obtained the probability of a positive result in each subject using logistic regression and expressed it in the form of a figure. Risk factors associated with IGRA positivity were investigated using logistic regression models, and the results were reported as odds ratios (ORs) and 95% confidence intervals (CIs). All tests of significance were 2-tailed, and a *p* value of less than 0.05 was considered statistically significant. All analyses were performed using SPSS software, version 23.0 (IBM, Armonk, NY, USA) and SAS 9.4 (SAS Institute, Cary NC).

## Results

### Baseline characteristics of the study population

**Fig. 1:**
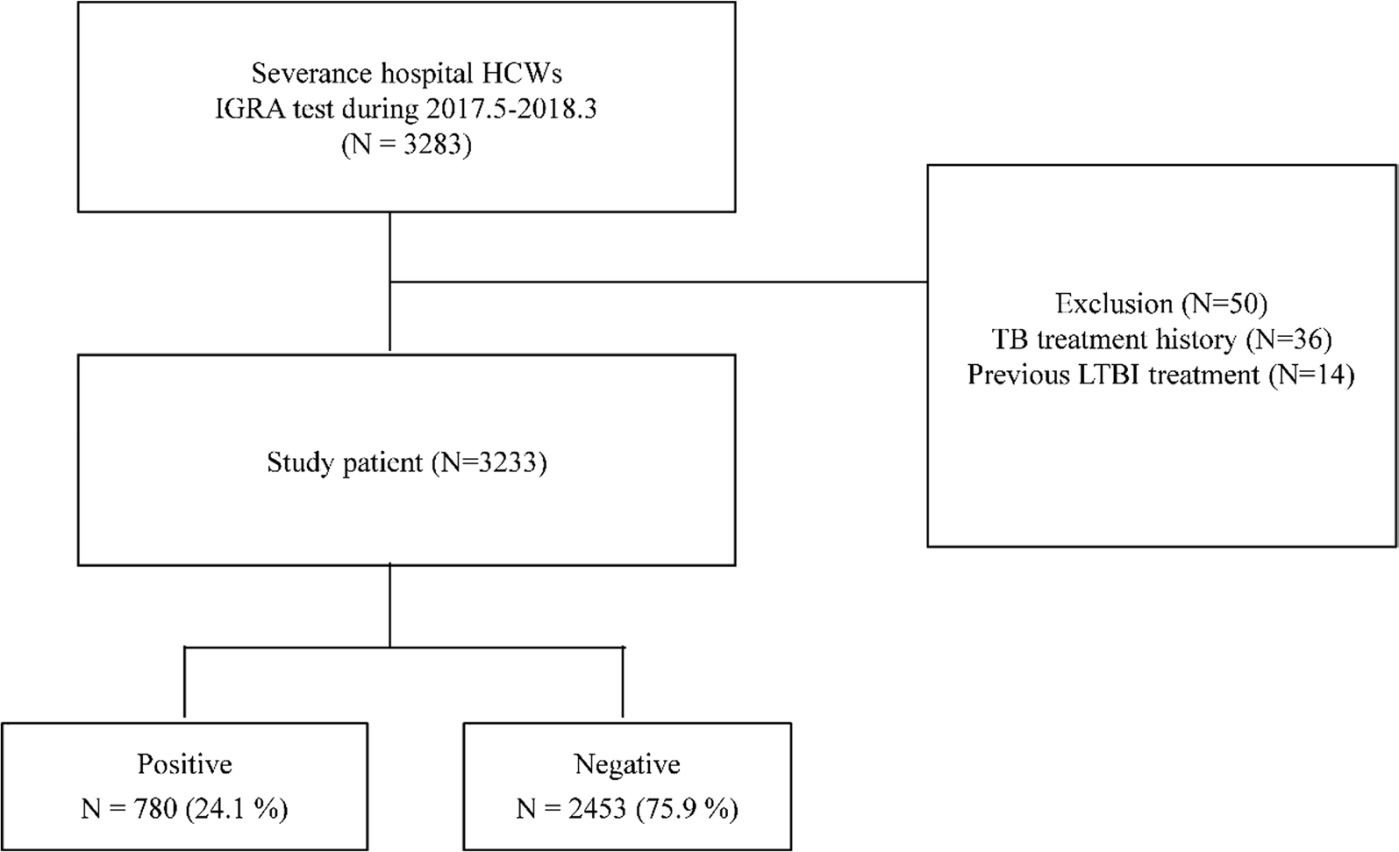
Flowchart of the HCWs in the study. *HCWs* health care workers; *IGRA* interferon-gamma release assay; *LTBI* latent tuberculosis infection

Overall, 3233 HCWs were analyzed after excluding 50 HCWs who had undergone previous anti-tuberculosis treatment or LTBI treatment (Fig. 1). We also excluded participants with presumptive active TB by chest x-ray and respiratory symptoms. There was no active TB case among our study participants.

Baseline characteristics of HCWs are shown in Table 1. Of the total 3233 HCWs, 2348 (72.6%) were female, and the median age was 38 (IQR 29–46). Occupationally, the proportion of heath aids, physicians, and nurses was similar. The working period was divided into groups ranging from less than 1 year to more than 30 years with the percentage of employees working for 10–20 years being the highest (30.1%). Forty-two HCWs (1.3%) reported healed TB lesion on chest x-ray. Overall, 780 of 3233 HCWs were IGRA positive (24.1%).

**Table 1:** Baseline characteristics of study population

	All participants (N = 3233)
Age (year), median (IQR)	38 (29–46)
20–29	835 (25.8)
30–39	904 (28.0)
40–49	999 (30.9)
50–59	449 (13.9)
≥ 60	46 (1.4)
Gender; female	2348 (72.6)
BMI (kg/m^2^), median (IQR)	22 (20–24)
Smoking status^a^	
Never smoker	2341 (72.4)
Ex-smoker (quit ≥ 1 year)	243 (7.5)
Current smoker	189 (5.8)
Health care professions	
Administrative	46 (1.4)
Technician^b^	449 (13.9)
Health aids^c^	999 (30.9)
Physicians	835 (25.8)
Nurses	904 (28.0)
Work duration (month), median (IQR)	
< 12 months	360 (11.1)
12 to < 60 months	722 (22.3)
60 to < 120 months	279 (8.6)
120 to < 240 months	667 (20.6)
240 to < 360 months	972 (30.1)
> 360 months	230 (7.1)
Comorbidities^d^	
Hypertension	210 (6.5)
Diabetes mellitus	85 (2.6)
Findings on chest X-ray^e^	
No active lung lesion	3063 (94.7)
Previously healed TB	42 (1.3)
Positive results of IGRA	780 (24.1)

Data are presented as numbers (percentages) unless otherwise indicated

^a^Total 2773 HCW, 460 missed data

^b^Technicians who perform radiological, laboratory and pathology testing

^c^Employees who provide physiotherapy and patient transfer services

^d^Total 2918 HCWs, 315 missed data

^e^Total 3105 HCWs, 128 missed data

### Prevalence of LTBI and predicted probability for IGRA positivity according to age

**Fig. 2:**
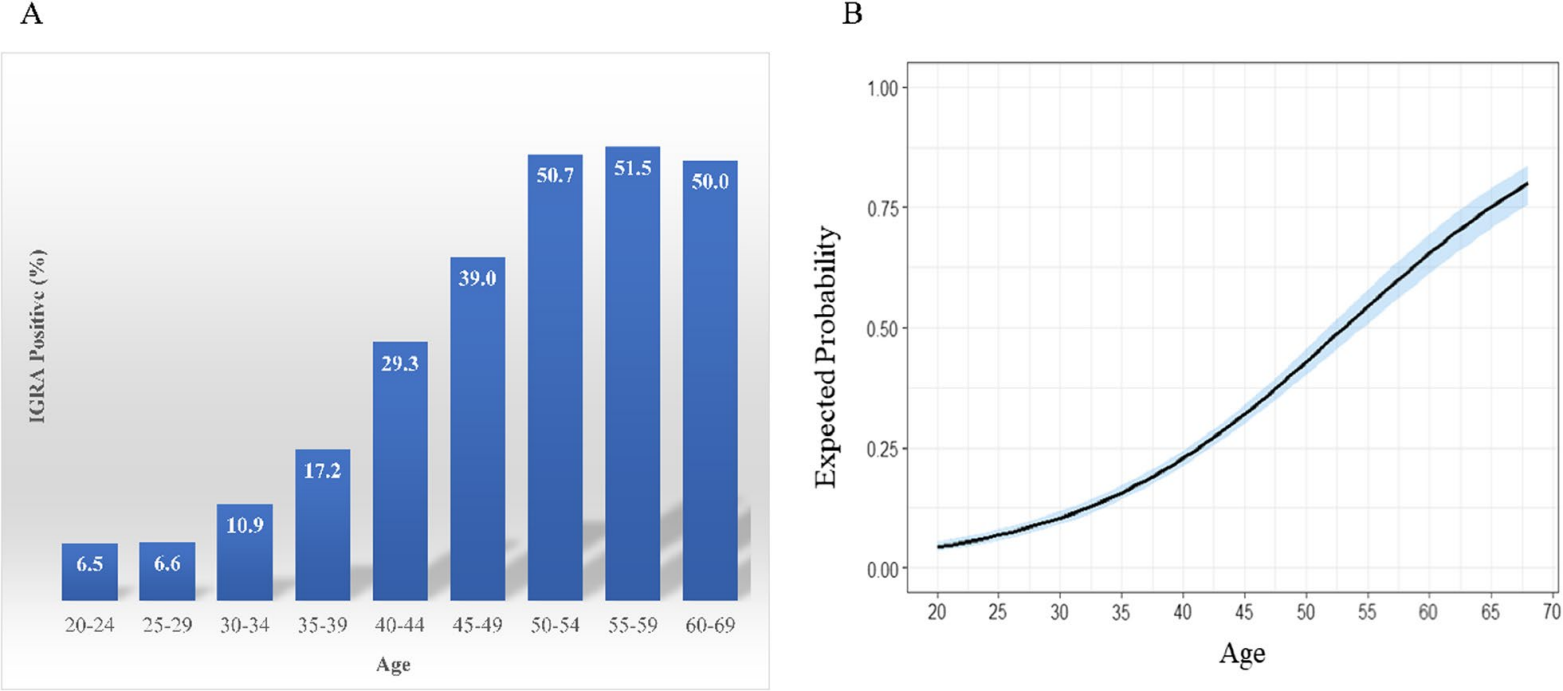
Prevalence of LTBI and predicted probability for IGRA positivity according to age. **A** IGRA positive rates according to age group. **B** Predicted probability for IGRA positivity according to age (*p* < 0.001)

Figure 2A shows the IGRA positive rate by age. Based on IGRA results, the prevalence of LTBI were 6.6% in the 20s, 14.4% in the 30s, 34.3% in the 40s, and around 50% in the 50s and 60s, respectively. As age increased, the likelihood of being positive on IGRA also increased, and the slope was 0.0929, which confirmed statistical significance (*p* < 0.001) (Fig. 2B). At age 40, the predicted probability of IGRA positivity was around 25% (Fig. 2B), and the results were confirmed to be similar to actual IGRA positive rates (Fig. 2A) (AUC = 0.749, diagnostic performance was good) [11] (Additional file 1: Fig. 1).

### Annual risk of tuberculosis infection (ARI)

**Fig. 3:**
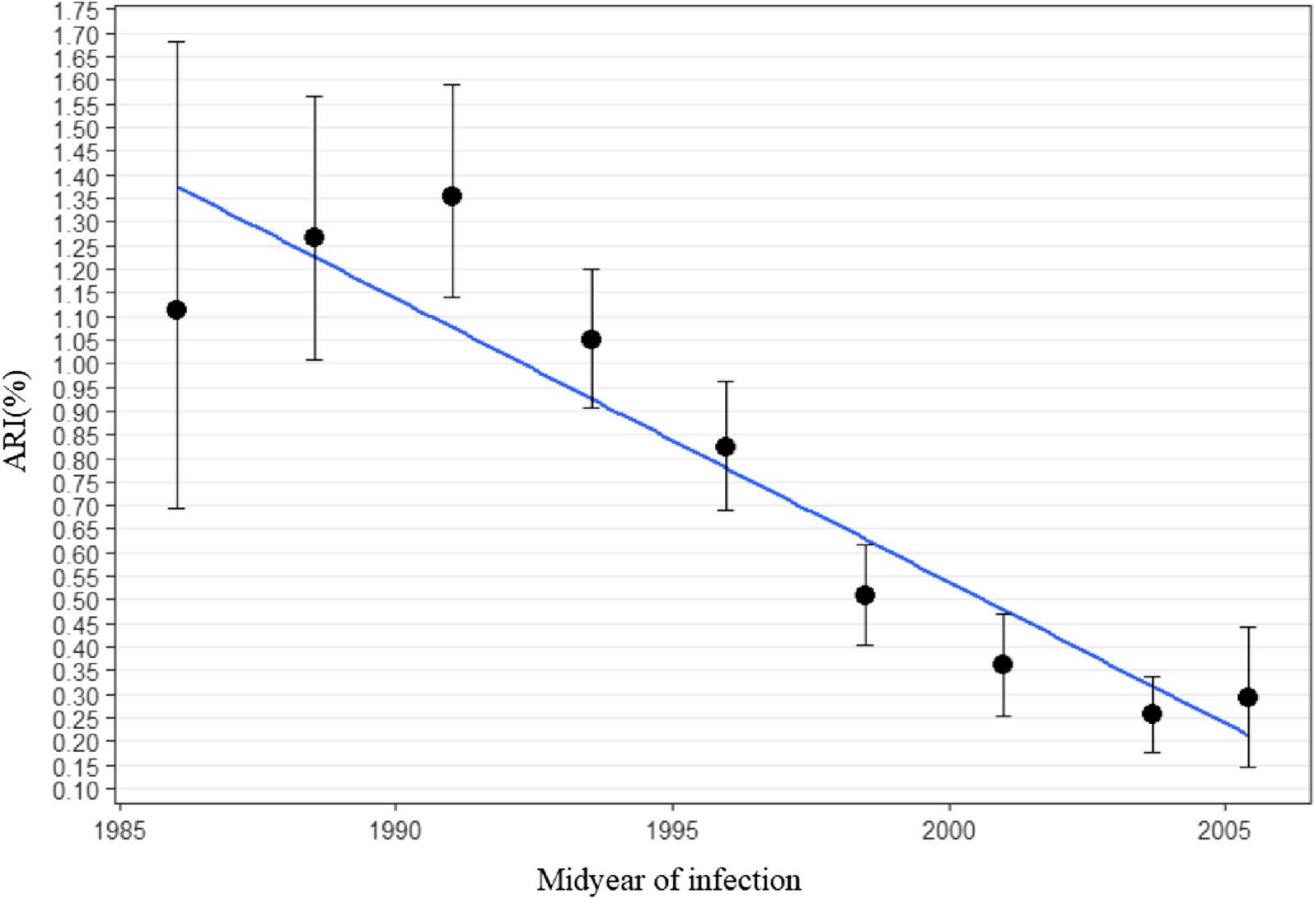
Annual Risk of TB Infection between 1986 and 2005

Table 2 and Fig. 3 shows the annual risk of tuberculosis infection between 1986 and 2005. The ARIs were 0.26–1.35% between 1986 and 2005. The risk has gradually decreased over the last two decades.

**Table 2:** Annual risk of tuberculosis infection

AGE	Number	MID_YEAR^a^	ARI (%)	95% CI
20–24	230	2005.4	0.29	0.15–0.44
25–29	605	2003.7	0.26	0.18–0.34
30–34	403	2001.0	0.36	0.26–0.47
35–39	501	1998.5	0.51	0.40–0.62
40–44	481	1995.9	0.82	0.69–0.96
45–49	518	1993.5	1.05	0.91–1.20
50–54	286	1991.0	1.35	1.14–1.59
55–59	163	1988.5	1.26	1.01–1.57
≥ 60	46	1986.0	1.11	0.69–1.68

^a^Midyear of TB infection (y) = 2017-mean age of each age group/2

### Risk factors of IGRA positivity

Table 3 shows the risk factors associated with positive IGRA results. According to univariable analysis, older age group, male gender, healed TB on chest x-ray, and longer working duration were risk factors for IGRA positivity. On the other hand, currently working in high-risk (TB related) departments was not a risk factor for IGRA positivity. Even though doctors and nurses face patients directly, IGRA positivity was lower than that among administrative staff, which is explained by the fact that the average age of doctors and nurses was younger than that of administrative staff (Additional file 2: Table 1). In multivariable analysis, older age, healed TB lesion on chest x-ray, and male gender were risk factors for IGRA positivity, whereas currently working in high-risk TB department was not.

**Table 3:** Factors associated with positive IGRA results

	IGRA			Total		Univariable				Multivariable			
	Negative	Positive				OR	95% CI		*p*	OR	95% CI		*p*
Age	N (%)	N (%)				1.096	1.086	1.107	< 0.001				
20–29	780 (93.4)	55 (6.6)		835		1				1			
30–39	774 (85.6)	130 (14.4)		904		2.259	1.612	3.166	< 0.001	2.25	1.593	3.18	< 0.001
40–49	656 (65.7)	343 (34.3)		999		7.031	5.165	9.570	< 0.001	5.95	4.282	8.268	0.0028
50–59	220 (49.0)	229 (51.0)		449		14.013	10.023	19.591	< 0.001	10.24	7.136	14.692	< 0.001
≥ 60	23 (50.0)	23 (50.0)		46		13.340	7.021	25.345	< 0.001	13.3	6.637	26.655	< 0.001
Gender, male	576/2453 (23.5)	308/779 (39.5)		884 (27.4)		2.299	1.931	2.737	< 0.001	1.56	1.243	1.968	< 0.001
Finding of CXR													
No active lung	2319 (75.0)	774 (25.0)		3093		1				1			
Healed TB	14 (33.3)	28 (66.7)		42		6.240	3.268	11.915	< 0.001	4.39	2.175	8.86	< 0.001
Occupation													
Administrative	212 (53.0)	188 (47.0)		400		1				1			
Technician^a^	155 (70.1)	66 (29.9)		221		0.473	0.334	0.671	0.6059	0.703	0.482	1.024	0.8625
Health-Aids^b^	642 (73.9)	227 (26.1)		869		0.447	0.348	0.575	0.9207	0.858	0.648	1.135	0.0454
Physician	212 (73.6)	76 (26.4)		288		0.403	0.290	0.561	0.4081	0.485	0.334	0.703	0.0040
Nurse	1232 (84.7)	223 (15.3)		1455		0.202	0.158	0.258	< 0.001	0.66	0.484	0.899	0.3822
Department													
High-risk^c^	521 (85.7)	87 (14.3)		608		0.443	0.348	0.566	< .0001	0.895	0.676	1.184	0.436
Others	1932 (73.6)	693 (26.4)		2625		1				1			
Working duration, months													
< 12 months	326 (90.6)	34 (9.4)		360		1							
12 to < 60 months	655 (90.7)	67 (9.3)		722		0.956	0.612	1.493	< .0001				
60 to < 120 months	236 (84.6)	43 (15.4)		279		1.675	1.027	2.733	0.0127				
120 to < 240 months	531 (79.6)	136 (20.4)		667		2.342	1.551	3.535	0.7556				
240 to < 360 months	583 (60.0)	389 (40.0)		972		6.116	4.152	9.010	< 0.001				
> 360 months	119 (51.7)	111 (48.3)		230		8.612	5.500	13.485	< 0.001				

Data are presented as numbers (percentages) unless otherwise indicated

*HCW* health care worker; *LTBI* latent tuberculous infection; *BMI* body mass index; *TB* tuberculosis; *CXR* chest X-ray; *IGRA* IFN-γ release assay; *IQR* interquartile range; *CI* confidence interval

^a^Technicians who perform radiological, laboratory and pathology testing

^b^Employees who provide physiotherapy and patient transfer services

^c^high risk department defined as those who are working at TB-related departments, such as respiratory department of ward and outpatient clinic, medical intensive care unit, emergency department, microbiology laboratory, and radiology department

## Discussion

In this study, using 3233 IGRA results of HCWs between 20 and 68 years of age, the overall prevalence of LTBI was 24.1%, and age was the strongest predictor of LTBI, rather than working in TB-related departments. The annual risk of TB infection ranged from 0.26 to 1.35% between 1986 and 2005, and its risk has gradually decreased over the last two decades.

ARI with *Mycobacterium tuberculosis* is known to be calculated from an observed prevalence of infection, approximating the incidence of infection. ARI for TB is one of the most important indicators for accessing its epidemiology in a population and is potentially informative about the transmission of TB within a community [12]. The main cause for the decline in ARI was a decreased prevalence of active TB in South Korea over 30 years, estimated from 443 to 167 cases per 100,000 population between 1985 and 2005, owing to economic growth and governmental effort to control TB via *Bacillus Calmette–Guérin* (BCG) vaccination, a TB notification system, and appropriate treatment through public health systems [3, 13]. Additionally, improvement on infection control at medical institutions is believed to have contributed to reducing ARI among HCWs.

To date, trends for TB prevalence and ARI have been documented in several countries, and most research has been based on national TST surveys [14–16]. In South Korea, Kim et al. reported that the prevalence of LTBI using TST in persons < 30 years of age decreased from 55.9% in 1965 to 30.8% in 1995. In conjunction with this change, the ARI of TB in persons < 30 years of age decreased from 5.3% in 1965 to 0.5% in 1995 [2]. The use of TST however, has a poor specificity due to its cross-reaction with BCG vaccine strain and nontuberculous mycobacteria. According to a study conducted in South Korea comparing TST and IGRA results according to TB infection risk [4], the agreement between TST and IGRA was not high, and IGRA was a better indicator of the risk of TB infection than TST in a BCG-vaccinated population. There have been attempts to estimate ARI using IGRA [17, 18]. Nishimura et al. used IGRA data from HCWs in a university hospital in Japan and reported estimated ARIs for TB of 0.156% in 1986 and 0.049% in 2004 [19]. In a recent prospective study of a population of 13,580 individuals in China, ARI was estimated by conversion of IGRA and TST [20]. The annual TB infection rate was suggested to be 3.1% based on IGRA conversion and 1.5% based on persistent positive results after IGRA conversion. Meanwhile, however, ARI based on TST conversion was 14.5%, suggesting of limitation of ARI estimation based on TST results. The study concluded that the use of IGRA was more accurate than TST when estimating ARI [20]. However, studies on ARI using IGRA results are lacking in South Korea. Among recent large-scale surveys, a survey of 2051 sample of the 2016 Korea National Health and Nutrition Examination Survey (KNHANES) showed that the overall prevalence of LTBI was 33.2% using TST results [3, 21], which was higher than our study results. Considering the false positivity of TST, we believed that our data using IGRA results would reflect more accurate LTBI prevalence and ARI in South Korea where BCG vaccination is mandatory.

Our study also suggested that LTBI was related to older age, healed TB lesion on chest x-ray, and male gender, and not to current occupation in high-risk TB department. Several previous studies reported that TB-related departments such as respiratory ward or clinic, medical ICU, and emergency department increased the risk of LTBI positivity as compared to other departments [22–24]. However, the majority of studies reporting such analyses were conducted in low-incidence settings. Contrarily, in countries with a relatively high TB burden, several studies have reported no significant differences in the LTBI positivity in HCWs working in TB-related department and other departments [25, 26]. The possible reasons for our results include a probability of casual contact with active TB patients in Korea, increased age regardless of working department, and adequate infection control in tertiary hospitals in our study setting.

Although the ARI for TB gradually decreased over 20 years in our study, LTBI prevalence and ARI among HCWs in South Korea were still higher than those in developed countries. This study also showed that the prevalence of LTBI was affected more by age-related risk exposure than working in a TB-related department. It is well known that exposure to TB at a young age and recent infection are major risk factors for the progression to active TB [27, 28]. Therefore, in South Korea, approaches to LTBI treatment among HCWs according to age should be differentiated due to a high likelihood of recent infections at a young age regardless of the department in which one is currently working.

This study has several limitations. First, we conducted this study at a single, tertiary hospital; hence, the findings may not be representative of HCWs in other hospital nor general population in South Korea. Second, in calculating ARI in this study, we used cross-sectional data rather than longitudinal data; hence, the accuracy of findings for old age may decrease given that the IGRA test scores wane as the age increases. Despite these limitations, to our knowledge, this is the first study in South Korea to report the estimation of ARI for TB based on IGRA results, and our study included large HCWs of different ages, working department, and working durations. Considering that age has a great impact on LTBI positivity, long-term follow-up studies are needed to identify how the progression from LTBI to active TB differs by age group.

## Conclusions

Our study showed that ARI in HCWs in South Korea has gradually decreased over the last two decades, but LTBI was still considerably prevalent. We also suggest that LTBI among HCWs might be greatly affected by age, rather than occupational exposure in intermediate TB burden countries, hence different LTBI treatment strategies by age group should be considered.

## Supplementary Information

**Additional file 1: Figure 1.** Predictive value of Age for IGRA positivity.

**Additional file 2: Table 1.** IGRA positivity according to occupation and working department.

## Data Availability

The datasets generated and analyzed during the study are available from the corresponding author on reasonable request.

## Abbreviations

TB: Tuberculosis
LTBI: Latent tuberculosis infection
ARI: Annual risk of TB infection
TST: Tuberculin skin test
BCG: *Bacillus Calmette–Guérin*
HCWs: Healthcare workers
IGRA: Interferon-gamma release assays
QFT-GIT: QuantiFERON® TB-Gold In Tube test
NTM: Nontuberculous mycobacteria
KNHANES: National Health and Nutrition Examination Survey
