# Supplementary material for: Decreased annual risk of tuberculosis infection in South Korean healthcare workers using interferon-gamma release assay between 1986 and 2005
Source: BMC Infect Dis. 2021 Nov 16;21:1161. doi: 10.1186/s12879-021-06855-5 (PMC8594200; doi:10.1186/s12879-021-06855-5)
Supplement: Supplementary file 1 — Additional file 1: Figure 1. Predictive value of Age for IGRA positivity. [file 12879_2021_6855_MOESM1_ESM.docx]

**Additional Figure 1. Predictive value of Age for IGRA positivity**


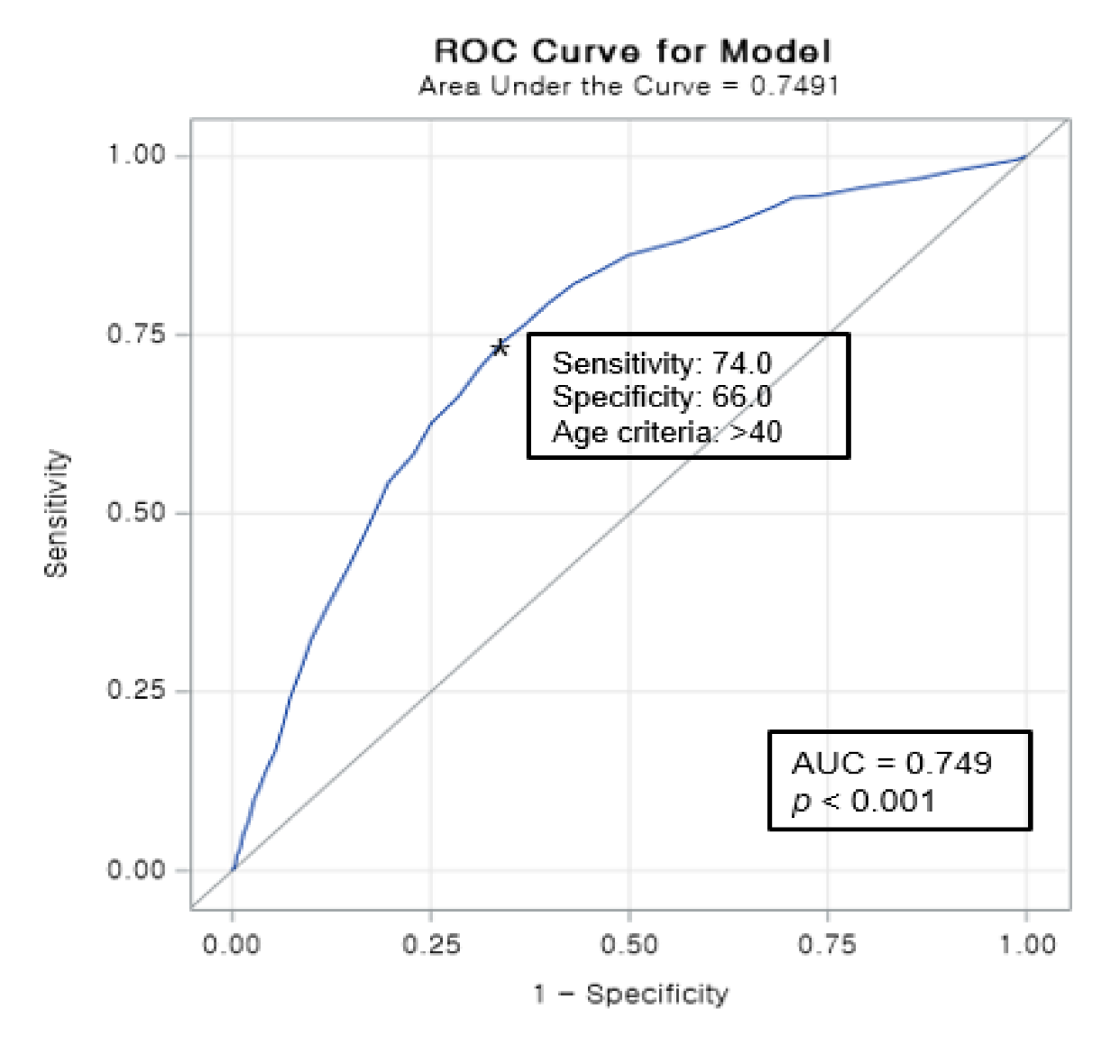


To determine the relationship between age and positivity of IGRA, a receiver operating characteristic (ROC) curves was generated and area under curve (AUC) was 0.749 by age cut off >40
