# Supplementary material for: Decreased annual risk of tuberculosis infection in South Korean healthcare workers using interferon-gamma release assay between 1986 and 2005
Source: BMC Infect Dis. 2021 Nov 16;21:1161. doi: 10.1186/s12879-021-06855-5 (PMC8594200; doi:10.1186/s12879-021-06855-5)
Supplement: Supplementary file 2 — Additional file 2: Table 1. IGRA positivity according to occupation and working department. [file 12879_2021_6855_MOESM2_ESM.docx]

Additional Table 1. IGRA positivity according to occupation and working department

| **Occupation** | **Department** | IGRA (+) | Mean Age | **Mean age** |
| --- | --- | --- | --- | --- |
| Administrative | High-risk | 0 (0.00%) | NA | 47.48±6.74 |
|  | Others | 188/400 (47.00%) | 49.31±5.46 |  |
| Technician | High-risk | 18/75 (24.00%) | 41.89±11.28 | 42.36±10.01 |
|  | Others | 48/146 (32.88%) | 48.42±5.71 |  |
| Health-Aids | High-risk | 21/70 (30.00%) | 44.86±12.19 | 38.22±10.35 |
|  | Others | 206/799 (25.78%) | 43.90±8.66 |  |
| Physician | High-risk | 9/55 (16.36%) | 45.89±9.06 | 43.31±11.39 |
|  | Others | 67/233 (28.76%) | 50.76±10.25 |  |
| Nurse | High-risk | 39/408 (9.56%) | 35.67±8.29 | 34.60±8.41 |
|  | Others | 184/1047 (17.57%) | 41.79±8.16 |  |

^a^ Technicians who perform radiological, laboratory and pathology testing.

^b^ Employees who provide physiotherapy and patient transfer services.

^c^ high risk department defined as those who are working at TB-related departments, such as respiratory department of ward and outpatient clinic, medical intensive care unit, emergency department, microbiology laboratory, and radiology department.
